# Respiratory effects of nusinersen treatment in pediatric patients with spinal muscular atrophy types 2 and 3

**DOI:** 10.1007/s00431-025-06475-0

**Published:** 2025-10-20

**Authors:** Mika Rochman, Dar Shay Levanon, Israel Amirav, Moria Be’er, Michal Cahal, Omri Besor, Aviva Fattal-Valevski, Moran Lavie

**Affiliations:** 1 Pediatric Pulmonology Unit, Dana-Dwek Children‘s Hospital, Tel Aviv Sourasky Medical Center, 6 Weizman Street, 6423906 Tel Aviv, Israel; 2 Gray Faculty of Medical and Health Sciences, Tel Aviv University, Tel Aviv, Israel; 3 Department of Family Medicine, Maccabi Healthcare Services, Tel Aviv, Israel; 4 Pediatric Neurology Institute, Dana-Dwek Children‘s Hospital, Tel Aviv Sourasky Medical Center, Tel Aviv, Israel

**Keywords:** Spinal muscular atrophy, Respiratory, Pulmonary function tests, Nusinersen, Restrictive lung disease

## Abstract

Spinal muscular atrophy (SMA) is a progressive neuromuscular disorder characterized by respiratory decline. While nusinersen improves motor function, its long-term respiratory effects in milder SMA types (2 and 3) remain unclear. This study evaluates pulmonary outcomes following 3 years of treatment. We retrospectively reviewed genetically confirmed SMA 2 and 3 pediatric patients treated with nusinersen (2017–2022). Data included demographics, non-invasive ventilation (NIV) and mechanical insufflation-exsufflation (MIE) use, body mass index (BMI), feeding method, scoliosis, and respiratory hospitalizations. Pulmonary function tests (PFTs) at baseline and at year 3 included percent-predicted forced vital capacity (ppFVC), FEV1/FVC ratio, peak cough flow (PCF), maximal voluntary ventilation (MVV), maximal inspiratory pressure (MIP), and maximal expiratory pressure (MEP). Included were 15 patients with type 2 and 13 patients with type 3 SMA. Their mean age at diagnosis was 3.5 ± 4 years and 13.8 ± 5.4 years at nusinersen initiation. One was lost to follow-up. At baseline, 6 patients used NIV and 8 used MIE. After 3 years, NIV use increased to 7/27 and MIE to 14/27. All 27 patients maintained oral intake and their BMI remained stable. ppFVC declined, most notably in year 3 (− 13.5%, *p* < 0.001). PCF, MVV, MIP, and MEP values remained stable or mildly improved, suggesting a slower decline or relative stabilization compared to natural progression.

*Conclusion*: Nusinersen may help preserve respiratory muscle strength and delay the need for ventilatory support among pediatric patients with SMA types 2 and 3. Although ppFVC declined, stability in other parameters supports a potential respiratory benefit.

**Table:** 

**What is Known:** • SMA causes progressive respiratory decline over time.	
**What is New:** • Nusinersen may preserve respiratory muscle strength in SMA.

**What is Known:**

• SMA causes progressive respiratory decline over time.

**What is New:**

• Nusinersen may preserve respiratory muscle strength in SMA.

## Introduction

Spinal muscular atrophy (SMA) is an autosomal recessive neuromuscular disease caused by homozygous mutations in the survival motor neuron (SMN) 1 gene leading to SMN protein deficiency and progressive motor neuron degeneration [1–3].

The classic phenotypic classification (types 0–4) is based upon age at onset and the highest motor milestone achieved (e.g., sitting, walking) [1, 2]. However, in the era of disease-modifying therapies, this classification has become less definitive for prognosis, and the SMN2 copy number is increasingly recognized as a key genetic modifier of SMA severity. The SMN2 copy number influences age at onset, motor milestone achievement, and disease progression, and is now more commonly used to guide clinical decision-making and anticipate treatment response.

The current study focuses upon SMA Type 2, which typically presents between 6 and 18 months and characterized by the ability to sit but not walk, and Type 3, which emerges between 18 and 36 months and presents with varying degrees of mobility. Both types involve progressive motor decline and weakness[1, 2, 4, 5]. The impact of muscle weakness on the patient's respiratory system has a significant influence upon disease progression. Respiratory muscle weakness contributes to restrictive lung disease, ineffective cough, increased infection risk, tachypnea, paradoxical breathing, and eventual respiratory failure[1, 5–7].

Current disease-modifying therapies, such as nusinersen (Spinraza) and others have been shown to improve motor and neurological function and milestone achievements in SMA patients [2, 4, 5, 8]. Short-term studies in SMA1 patients suggest that nusinersen stabilizes respiratory function, thereby preventing expected decline, although long-term effects remain unclear [4, 9–12]. Data on the milder forms SMA types 2 and 3 are limited, and they primarily focus upon short-term pulmonary function outcomes in adults, with mixed findings regarding ventilatory support [13]. We therefore conducted this study to evaluates pulmonary outcomes in pediatric patients with SMA type 2 and 3 who are under nusinersen treatment.

## Material and methods

This is a retrospective analysis of respiratory data for pediatric patients with SMA types 2 and 3 treated with nusinersen[14]. This study adhered to the principles outlined in the Declaration of Helsinki. Ethical approval was granted by the ethics committees of Tel Aviv Sourasky Medical Center (0873–17-TLV). All participants had confirmed genetic mutations in the SMN1 gene, and treatment decisions were based on confirmed pathogenic SMN1 variants together with a clinical presentation consistent with SMA type 2 or type 3 [1, 2, 5]. SMN2 copy number, when available, was obtained from the medical records. They received nusinersen treatment throughout the study period and were routinely evaluated from 2017 to 2022 at our SMA multidisciplinary clinic. Pre-treatment assessments were defined as the closest available measurements within 6 months prior to nusinersen initiation, and post-treatment assessments were those performed at approximately 36 months (± 3 months) after treatment initiation. All clinical data, including pulmonary function tests and chest X-rays, were retrieved from the institutional database, which contains the exact dates for each evaluation. The clinical data extracted from their medical records included age, sex, SMA subtype, age at diagnosis and at treatment initiation, body mass index (BMI), feeding route, respiratory hospitalizations, formal radiological interpretation of chest X-ray findings, and presence of scoliosis. Also recorded were respiratory parameters, such as the use of assisted ventilation (invasive/non-invasive), permanent (> 16 h/day) or intermittent (< 16 h/day) and mechanical insufflation-exsufflation (MIE) including cough assist. Pulmonary function tests included forced vital capacity (FVC), forced expiratory volume in the first one second to forced vital capacity (FEV1/FVC) to assess lung function and maximum voluntary ventilation (MVV), maximum expiratory pressure (MEP), and maximal inspiratory pressure (MIP) to evaluate respiratory muscle strength. Cough effectiveness was measured via peak cough flow (PCF).

### Statistical analysis

The demographic variables, clinical respiratory variables, comorbidities, and lung function variables (PCF, MIP, MEP, FVC, and FEV1/FVC) were analyzed by descriptive statistics, including means and standard deviations (SDS) for normally distributed data or medians and interquartile ranges for non-normally distributed data. Normality was assessed by the Shapiro–Wilk test. Within-subject comparisons were conducted with the Wilcoxon signed-rank test due to violations of normality assumptions. Additionally, a linear mixed model was employed to assess pulmonary function trends over time, accounting for individual variations in the age at treatment initiation, baseline pulmonary function, and the natural progression of the disease. Statistical significance was set at *p* < 0.05, and all analyses were performed using R (R Foundation for Statistical Computing, Vienna, Austria).

## Results

A total of 28 patients (16 males, 12 females) were originally included, of whom 15 (54%) had SMA type 2 and 13 (46%) had SMA type 3. SMN2 copy number was available for 18 patients, with the following distribution: among those defined as SMA type 2, one patient had zero copies, one had 1 copy, two had 2 copies, and six had 3 copies. Among those defined as SMA type 3, one patient had zero copies, one had 1 copy, three had 3 copies, and three had 4 copies.

In total, across the entire cohort, there were 2 (7%), 2 (7%), 2 (7%), 9 (32%) and 3 (11%) patients with zero, 1, 2, 3, and 4 SMN2 copies respectively. The two patients recorded with zero SMN2 copies most likely represent technical artifacts rather than a true biological absence of SMN2, given the known methodological limitations of MLPA/qPCR assays and the structural complexity of the SMN locus [15]. The average age of the cohort at diagnosis was 3.5 (± 4.03) years and 13.8 (± 5.42) years at initiation of nusinersen.

### Baseline

At baseline, 6 (21.4%) patients, all type 2 SMA, were ventilated intermittently, all by non-invasive ventilation (NIV). Eight (28.5%) patients used MIE, 4 on a regular basis and 4 during exacerbations. All of the study patients were fed orally, with a mean BMI (SDS) of −0.58 (± 2.8). Scoliosis was present in 22 patients, 16 of whom had undergone prior surgical repair. The average number of respiratory hospitalizations in the 3 years preceding treatment was 0.48 (0–6). Chest X-rays revealed that 2 patients had a bell-shaped chest, with no other significant pathological findings reported on those imaging studies. Transcutaneous CO₂ or arterial blood gases were available for 13 patients, with a mean value of 40.6 ± 5.7 mmHg (2 of the 3 patients who had values above 45 mmHg were on NIV). Sleep studies were performed in 10 patients, revealing obstructive sleep apnea (OSA) in 3 and oxygen desaturation in 3, while none had hypoventilation, defined as nocturnal TcCO₂ > 50 mmHg for more than 25% of total sleep time or sustained daytime PaCO₂ > 45 mmHg, consistent with established clinical criteria in neuromuscular disorders[16].The baseline means for each pulmonary function test (PFT) were: FVC 79% predicted, FEV1/FVC 102% predicted, peak cough flow 285 L/min, MVV 62.1%, and MEP 50 cmH20. The MIP was ≥ 50 cmH20 in 14/24 patients.

### Post-treatment

One patient was lost to follow-up, leaving a total of 27 patients for final assessment.

**Fig. 1:**
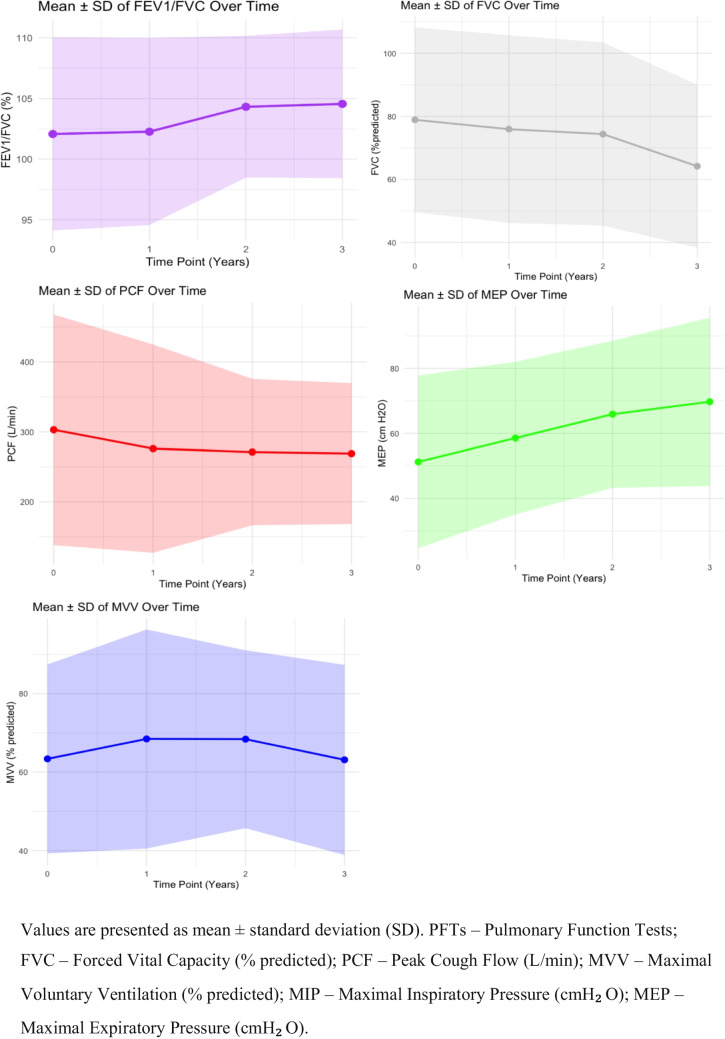
Mean and standard deviation of pulmonary function test values over 3 years (line plot)

Ventilatory support, specifically intermittent NIV, was initiated in one patient due to OSA during follow-up, bringing the total to 7 of the 27 who were using NIV, 6 of them with SMA type 2 and 1 with SMA type 3. MIE was already being used by 8 patients at baseline and initiated in 6 additional patients to reach a total of 14 patients. All of the study patients maintained oral intake, with no need for gastrostomy. The BMI SDS remained stable (−0.58 to −0.82; p = ns). Scoliosis was newly diagnosed in 3 patients, resulting in 25 patients with scoliosis at study closure 3 additional patients underwent scoliosis surgery during the study period, added to the 16 patients had undergone scoliosis surgery before study entry. The average number of respiratory hospitalizations decreased to 0.11 (0–3) hospitalization/3 years (p = 0.057) (Table 1). There was a notable reduction in hospitalizations but it did not reach a level of significance. The main findings on chest X-rays were a bell-shaped chest in 8 patients and pulmonary atelectasis in 3 patients with no other significant pathological findings reported on those imaging studies. Post-treatment, transcutaneous CO₂ or arterial blood gases were available for 4 patients, with a mean value of 35.9 ± 9.2 mmHg. Sleep studies were performed in 18 patients and showed hypoventilation in 1 and OSA in 5. Two of the latter patients were recommended to start NIV based upon these findings, and NIV was initiated in one while the other declined. At 3 years, the mean FVC declined to 64% predicted compared to 79% predicted at baseline. A linear mixed model revealed a 2.9% decrease in the first year, a 3.3% decrease in the second year, and a 13.5% decrease in the third year (*p* < 0.001). FEV1/FVC remained stable at 105% predicted compared to 102% predicted at baseline, with a slight increase of 2.4% and 2.6% observed in the second and third years, respectively. Peak cough flow decreased slightly to 269 L/min compared to 285 L/min at baseline, with no significant change over time (*p* = ns). MVV remained stable throughout the study period. MEP increased to 70 cmH₂O compared to 50 cmH20 at baseline, with the linear mixed model indicating increases of 10.4, 18.5, and 21.5 cmH₂O in the first, second, and third years, respectively. MIP improved significantly by year 3 (*p* < 0.01), with 20 patients achieving MIP > 50 cmH₂O. In summary, although FVC declined, FEV1/FVC, PCF, MVV, MEP, and MIP remained stable, with some measures demonstrating mild improvement (Fig. 1).

**Table 1:** Clinical and respiratory data at baseline and after 3 years of nusinersen treatment

Clinical and respiratory data	Baseline (n = 28)	After 3 years (n = 27)
Use of NIV, n	6 (21.4%)	7 (26%)
Use of MIE, n	8 (28.5%)	14 (51.8%)
Daily	4 (10.7%)	5 (18.5%)
During exacerbation	4 (14.2%)	3 (11%)
Respiratory hospitalization, range/3yrs	0.48 (0–6)	0.11 (0–3)
Oral feeding, n	28 (100%)	27 (100%)
BMI (mean ± SDS)	−0.58 ± 2.8	−0.824 ± 3.0
Scoliosis, n	22 (78%)	25 (92.6%)

NIV—non-invasive ventilation, MIE—mechanical insufflation-exsufflation, BMI – body mass index

## Discussion

The present study presents the respiratory effects of nusinersen treatment in pediatric patients with SMA types 2 and 3 over a 3-year period. While the therapeutic focus of nusinersen has predominantly been upon motor function, its impact on respiratory outcomes is no less important for patient management. Our findings indicate a stabilization of respiratory muscle strength in parallel with progressive restrictive lung disease characterized by a decline in FVC but preservation of MIP, MEP, and PCF.

The marked FVC decline in year 3 (− 13.5%, p < 0.001), following smaller decreases in years 1 (− 2.9%) and 2 (− 3.3%), likely reflects progressive scoliosis, respiratory muscle weakness, loss of ambulation, and other disease-related changes, consistent with findings in untreated cohorts [1, 6, 17]. In the first 2 years post-treatment, our results align with updated iSMAc data from Trucco et al. (2024)[18], which show that nusinersen slows respiratory decline in type 2 and non-ambulant type 3 SMA compared with historical controls, though declines persist in those with established scoliosis. Other real-world studies, such as those by Chacko et al. [19, 20] and Côté et al. [21], have reported stable FVC or early improvements in type 2 patients [22], while Aldukain et al.[13] and Wurster et al. [23] describe phenotype and timing dependent variability in respiratory outcomes.However, the lack of association between FVC decline and stable/increasing MIP/MEP suggests that nusinersen may mitigate respiratory muscle weakness while having only a limited impact upon thoracic structural decline (e.g., scoliosis progression, chest wall rigidity). This aligns with the findings of Gómez-García de la Banda et al. [5], who reported that respiratory muscle strength was preserved despite declining lung volumes in nusinersen-treated patients.

Notably, MEP improved to 70 cmH2O (*p* < 0.01), which may reflect enhanced expiratory muscle function critical for airway clearance. The stability of MVV over time (ranging from 62.1% to 62%) further supports compensatory mechanisms despite restrictive pathophysiology. Recent systematic reviews have also suggested that early treatment initiation may maximize pulmonary benefits, given that younger patients exhibit less severe declines in FVC and greater improvements in respiratory muscle strength compared to older patients [1, 13].

After 3 years of follow-up, NIV was initiated in only 1 of the 21 patients who were not using ventilatory support at baseline due to OSA, bringing the total to 7 patients on NIV, 6 of them among the 15 patients with SMA type 2 and only 1 among the 13 SMA Type 3 patients. This represents the lower limit of natural history data predicting NIV use in 39–45% of patients with SMA type 2 and 5–9% of those with SMA type 3 [17, 24]. These results are not as favorable as those reported by Scheijmans et al. [25], where 41 out of their 46 nusinersen-treated patients with SMA types 2 and 3 remained NIV-free. The delayed need for ventilatory support in our cohort may reflect the treatment’s positive role in stabilizing respiratory muscle function. As observed by Panagiotou et al.[26], our findings contribute to the growing body of evidence suggesting that while nusinersen may postpone the onset of NIV requirement, it does not entirely prevent it. Furthermore, several systematic reviews [19, 23] have demonstrated that nusinersen can reduce sleep-disordered breathing and enhance peripheral motor performance, both of which may further contribute to delaying ventilatory support.

Additional noteworthy clinical findings in our study was the preservation of oral feeding without the need for gastrostomy insertion, stable BMIs, and a reduction in hospitalization rates, all contributing to a reduced respiratory disease burden. These results expand upon the sparse evidence currently available in the literature [27–29] and are consistent with the findings of Gaboli et al., who studied nusinersen-treated pediatric SMA type 2 and 3 patients and reported significant improvements in FVC and FEV₁ z-scores in type 2 patients over a two-year period, as well as preservation of oral feeding without gastrostomy [22].

Progressive spinal deformity was also prevalent in our study, with the prevalence of scoliosis reaching as high as 93%, and with 70% of those patients requiring and undergoing surgery. These findings likely contributed to the accelerated FVC decline that reduced chest wall compliance recorded in year 3, consistent with the findings of Kaufmann et al.[30]. This underscores the need for concurrent orthopedic interventions, given that nusinersen alone does not halt structural thoracic decline. Multidisciplinary management approaches integrating orthopedic care are clearly essential for mitigating scoliosis-related respiratory deterioration.

This study has several limitations, including its retrospective design, small sample size (n = 28) due to the rarity of SMA, and the absence of a historical treatment-naïve control group. Although we explored the possibility of creating such a cohort, differences in historical patient management and the lack of systematic respiratory follow-up precluded a reliable comparison.

However, the alignment of our findings with other cohorts strengthens their validity.

In conclusion, treatment of SMA types 2 and 3 with nusinersen may stabilize respiratory muscle strength and delay NIV dependence, despite progressive restrictive lung disease. The decline of FVC in year 3 of our study, however, highlights the persistent impact of thoracic structural deterioration, necessitating a multidisciplinary approach to patient care. Integrating pulmonary monitoring, orthopedic management, and early interventions to provide airway clearance and ventilatory support are essential for optimizing outcomes. While our findings suggest that nusinersen provides meaningful respiratory benefits, further prospective research is needed to clarify its long-term impact on pulmonary function and disease progression in pediatric patients with SMA types 2 and 3.

## Data Availability

Data supporting the findings of this study are available from the corresponding author upon request.

## Abbreviations

SMA: Spinal muscular atrophy
NIV: Non-invasive ventilation
MIE: Mechanical insufflation-exsufflation
BMI: Body mass index
PFTs: Pulmonary function tests
ppFVC: Percent-predicted forced vital capacity
FEV1/FVC: Forced expiratory volume in 1 s/forced vital capacity
PCF: Peak cough flow
MVV: Maximal voluntary ventilation
MIP: Maximal inspiratory pressure
MEP: Maximal expiratory pressure
